# Dietary caffeine and its negative link to serum Klotho concentrations: evidence from the National Health and Nutrition Examination Survey

**DOI:** 10.3389/fnut.2024.1497224

**Published:** 2024-12-11

**Authors:** Haimeng Wu, Ping Lu

**Affiliations:** Department of Endocrinology, Fuwai Central China Cardiovascular Hospital, Zhengzhou, China

**Keywords:** Klotho, caffeine, dietary, NHANES, aging

## Abstract

**Background:**

This is the initial investigation assessing the association between caffeine consumption through diet and circulating Klotho concentrations, with Klotho being recognized as a key biomarker of healthspan and aging.

**Methods:**

This cross-sectional analysis utilized data from 11,169 adults who participated in the National Health and Nutrition Examination Survey (NHANES). Caffeine consumption was evaluated using 24-h dietary recall interviews by trained professionals, and serum Klotho concentrations were measured via an enzyme-linked immunosorbent assay (ELISA). Generalized linear models and threshold effect analysis were employed to examine the relationship between caffeine intake and serum Klotho concentrations. Interaction tests and subgroup analyses were conducted to identify potential effect modifiers.

**Results:**

After controlling for covariates, a negative correlation was observed between dietary caffeine consumption and serum Klotho concentrations, with each additional 100 mg of dietary caffeine consumption, Klotho decreased by 3.40 pg./mL (95% confidence interval [CI]: −5.73, −1.07). Participants in the fourth quartile of dietary caffeine consumption showed a 23.00 pg./mL reduction in serum Klotho concentrations (95% CI: −39.41, −6.58) compared to individuals in the first quartile. Threshold effect analysis revealed a threshold point corresponding to natural log-transformed caffeine value >3.74 (equivalent to ~41 mg/day), above which Klotho levels demonstrated a more pronounced decline. Subgroup analyses indicated that this association was more significant in participants with sedentary activity >480 min and without hypertension.

**Conclusion:**

Our study reveals a significant, dose-dependent negative association linking caffeine intake with serum Klotho concentrations in the United States adults aged 40–79 years, with potential thresholds beyond which the effects become more pronounced. Additional studies are required to verify these results and investigate the underlying biological processes involved.

## 1 Introduction

Caffeine ranks among the most commonly consumed psychoactive substances globally (1), predominantly sourced from coffee, energy drinks, and tea. Its health-related benefits and risks have been extensively studied (2). Prior studies have suggested that coffee consumption may reduce mortality risk (3, 4), decrease Parkinson’s disease risk (5), and have anti-aging effects (6, 7). Coffee contains various antioxidants, including caffeine, diterpenes, and polyphenols (8), which contribute to its noted health benefits. Evidence suggests that consuming up to 400 mg of caffeine daily does not present significant health risks for adults (2, 9). However, excessive caffeine intake may lead to insomnia, anxiety (10), increased risk of osteoporosis (11), and digestive issues (12). These mixed findings highlight the need for continued research on caffeine’s biological impact.

Klotho, a multifunctional protein, has gained increasing attention due to its connections to aging and longevity (13–15). Originally recognized for its role in promoting longevity in mice (13), Klotho operates in conjunction with fibroblast growth factors as a co-receptor and plays vital roles in regulating mineral metabolism, oxidative stress, and inflammation (14, 16–18). The protein that the Klotho gene encodes is known to exist as a transmembrane protein and is primarily found in the kidneys, parathyroid glands, and brain, with three known isoforms (19). Our research focuses on *α*-Klotho, a form capable of being released into circulation in its soluble form (20). In humans, elevated Klotho levels are associated with improved mental performance, renal protection, and reduced risk of age-related diseases (21–24), making it a key biomarker of healthspan and aging (25). Klotho protein expression decreases with age in both animals and humans (25, 26) and is influenced by factors like fibroblast growth factor 23, estrogen deficiency, oxidative stress, and angiotensin II (27, 28).

Emerging evidence suggests that dietary components may influence Klotho levels by modulating inflammatory pathways, oxidative stress responses, and cellular aging processes (29, 30), which are potential targets of caffeine’s bioactivity. Given that caffeine can exhibit both antioxidant and pro-oxidant properties depending on experimental conditions (31), it is plausible that caffeine might impact Klotho expression either directly, by altering oxidative stress levels, or indirectly, by influencing related metabolic or inflammatory pathways. Furthermore, studies have shown that caffeine can inhibit DNA repair (32), shorten telomeres (33), and may have carcinogenic effects (34). These effects, which are crucial in cellular aging, could provide additional mechanisms through which caffeine might potentially impact Klotho levels and aging.

To our knowledge, research on the connection between caffeine intake and Klotho concentrations is scarce, marking this as an emerging topic within aging research. In a large NHANES study of 4,780 women, Jason J. Liu identified a notable link between higher coffee intake and longer telomere length. However, the linear trend observed for total dietary caffeine consumption with telomere length (P for trend <0.05) vanished once adjustments for overall coffee consumption were made (P for trend = 0.37) (35). Similarly, Larry A. Tucker’s study, also using NHANES data, reported a negative correlation between caffeine intake and telomere length (*p* = 0.0005), while coffee intake was positively correlated (*p* = 0.0013) (36). These differing findings on caffeine and coffee suggest that caffeine’s impact on aging biomarkers could differ from that of coffee itself, prompting us to further investigate its specific association with the anti-aging protein Klotho. Clarifying this relationship could have important implications for refining dietary guidelines and interventions aimed at promoting healthy aging.

Our research seeks to evaluate the link between caffeine consumption with Klotho concentrations in a clinical population using NHANES data, while considering the impact of covariates such as sex, age, race, sedentary activity, and comorbidities. This study aims to uncover how caffeine might affect aging processes and related health outcomes. Given the widespread consumption of caffeine and the pivotal role of Klotho in aging, these findings could inform novel strategies to improve quality of life and promote longevity.

## 2 Materials and methods

### 2.1 Population sample

Organized under the Centers for Disease Control and Prevention, NHANES is a continuous program that gathers detailed nutrition and health information from the United States population by collecting extensive data via questionnaires, laboratory assessments, and physical examinations. NHANES assesses a nationally representative cohort of nearly 5,000 individuals annually, selected across various counties throughout the United States, with 15 counties visited per year. NHANES uses a stratified, multistage probability sampling approach (37). The approval for NHANES protocol was granted by the National Center for Health Statistics (NCHS) Research Ethics Review Board, and all participants provided documented consent in writing. Additional information on NHANES is available on its website.^1^

**Figure 1:**
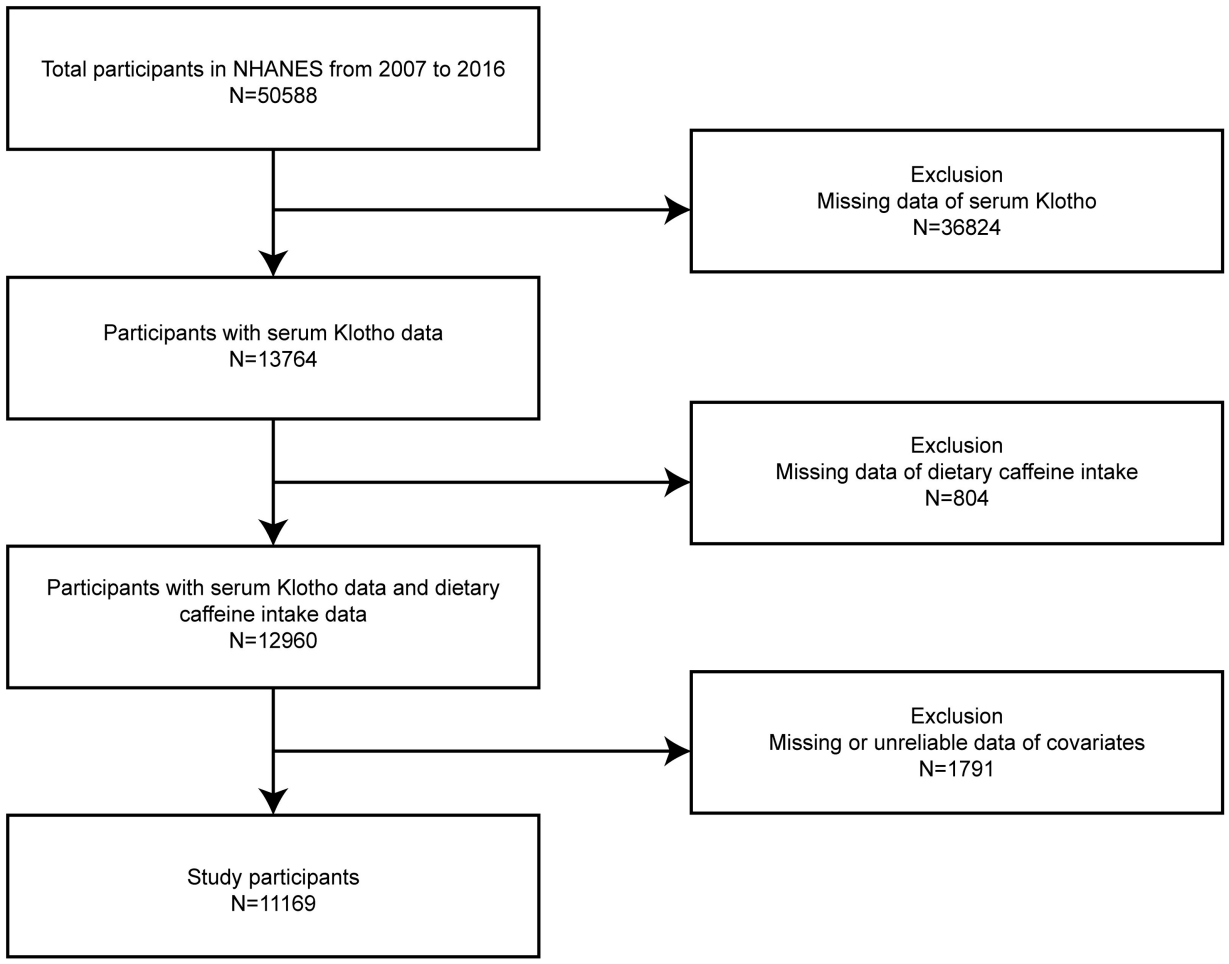
Flowchart illustrating the selection process of participants from the NHANES 2007–2016 dataset.

Our study participants were selected across the consecutive NHANES years spanning 2007 through the 2016 cycle. Individuals who had fully available serum Klotho data, precise dietary recalls, and relevant confounding variables were included (*n* = 11,169). From the initial pool of 50,588 individuals aged 40–79 years, we excluded 36,824 individuals who were missing serum Klotho data. An additional 804 participants with inadequate dietary recalls and 1,791 with insufficient covariate information were also removed. Figure 1 illustrates the detailed procedure for participant exclusion and inclusion.

### 2.2 Evaluation of dietary caffeine consumption

In this study, caffeine consumption (mg/day) served as the independent variable, with information obtained through the initial 24-h dietary recall which was administered at the Mobile Examination Center, where participants used standardized measurement guides (e.g., bowls, cups, spoons) to report food quantities accurately. Interviewers received rigorous training and conducted computer-assisted interviews. Caffeine consumption was estimated utilizing the United States Department of Agriculture’s Food and Nutrient Database for Dietary Studies (38). The dataset did not include dietary supplements. Caffeine consumption data were derived from a single 24-h dietary recall, which may introduce limitations such as potential memory bias and lack of dietary variability, potentially impacting the accuracy of intake estimates.

### 2.3 Assessment of serum Klotho concentrations

Serum Klotho concentrations from NHANES participants were measured with an ELISA kit (IBL International, Japan). To maintain accuracy, results from two duplicate analyses were averaged. Samples were stored at −80 degrees Celsius before being processed at the Northwestern Lipid Metabolism and Diabetes Research Laboratory (University of Washington). The assay had a sensitivity of 4.33 pg./mL, with a mean serum Klotho concentration of 698.0 pg./mL. More detailed methods for the Klotho assay are available on the NHANES website.

### 2.4 Covariates adjustment

Drawing from previous research (25, 39–48), 14 potential covariates related to serum Klotho concentrations were included: sex, age, race, family poverty income ratio (PIR), education, alcohol consumption, serum cotinine, body mass index (BMI), estimated glomerular filtration rate (eGFR), hypertension, diabetes, coronary heart disease (CHD, cancer, and sedentary activity. Covariates, including both continuous and categorical variables, were collected via questionnaires, physical examinations, and lab tests. The study focused on participants aged 40–79, given that Klotho concentrations had been measured within that age range. Race included categories of Mexican American, Non-Hispanic White, Non-Hispanic Black, or Other. The classification of educational attainment included < high school, high school, and college or above. Alcohol consumption was defined as ≥12 or < 12 drinks per year based on questionnaire data. Using the Chronic Kidney Disease Epidemiology Collaboration equation,^2^ eGFR (mL/min/1.73 m^2^) was derived and categorized into <90 and ≥ 90 mL/min/1.73 m^2^ in subgroup analysis. Data on hypertension, diabetes, CHD, cancer, and sedentary activity were all derived from questionnaire data. Detailed descriptions of the measurement techniques are available on the NHANES.

### 2.5 Statistical analysis

Serum Klotho concentrations were categorized into quartiles. Due to non-normal distribution, continuous data are summarized using the median and interquartile range (IQR). The Kruskal-Wallis rank sum test was applied for intergroup comparisons. Categorical data are shown as percentages, with statistical comparisons made utilizing the chi-square test. These descriptive analyses were conducted unweighted.

In this study, dietary caffeine consumption, being non-normally distributed, was expressed in mg/day and ln-transformed to approximate normality. To assess the relationship of dietary caffeine consumption (per 100 mg/day and ln-transformed) and serum Klotho concentrations, we employed a generalized linear regression model, with controlling for covariates. Three models were constructed: Model 1 without adjustments, Model 2 adjusting for sex, race, and age, and Model 3 with full covariate adjustment (sex, race, age, PIR, education, BMI, eGFR, alcohol consumption, serum cotinine, hypertension, diabetes, CHD, cancer, and sedentary activity). Using the first quartile as the reference, dietary caffeine consumption was divided into quartiles to examine potential nonlinear relationships. The median consumption of each quartile was analyzed as a continuous variable to assess linear trends. Additionally, smooth curve fitting was applied to explore nonlinear dose–response relationships and threshold effect analysis was conducted to explore potential threshold points in the dose–response relationship between dietary caffeine intake and serum Klotho concentrations. A two-segment linear regression model was employed to identify significant threshold points, with separate regression coefficients estimated for caffeine intake levels below and above the identified threshold. Interaction tests and subgroup analyses were conducted to examine associations across various covariates. All analyses, including fully adjusted regression models (Model 3), smooth curve fitting, threshold effect analysis, interaction tests, and subgroup analyses, were conducted with full covariate adjustments and applied NHANES-recommended dietary sample weights to ensure national representativeness of the study population.

Statistical analyses were carried out with EmpowerStats^3^ (version 4.2) and R software^4^ (version 4.2.0). Statistical significance was defined as a *p*-value <0.05 (two-tailed).

## 3 Results

### 3.1 General characteristics of the study population

Baseline characteristics of participants across Klotho quartiles are shown in Table 1. Of the 11,169 individuals included, 5,478 were male (49.05%) and 5,691 were female (50.95%). The median age was 58 years (IQR: 49–66). Over half of the participants had completed college or higher education, and nearly 50% identified as non-Hispanic White. Daily caffeine consumption varied from 0 to 4,530 mg, with a median of 120.00 mg/day (IQR: 29.00–246.00). At 800.70 pg./mL (IQR: 654.10–990.00), the serum Klotho concentration’s median was recorded. Significant differences were found across Klotho quartiles for nearly all variables, except BMI and sedentary activity. In the top Klotho quartile, individuals were more frequently female (56.05%) and younger (median age 56 years; IQR: 48–64). A higher percentage had completed college or above (53.72%) and reported higher income levels (median PIR: 2.27). These individuals consumed less alcohol, had normal eGFR (median: 93.15 mL/min/1.73 m^2^), and were less likely to have hypertension, CHD, or cancer. Participants in this quartile also tended to have lower caffeine consumption.

**Table 1:** Baseline demographic and clinical characteristics of participants in the NHANES study by Klotho quartiles (*N* = 11,169).

Variables	All *N* = 11,169 (100%)	Quartiles of serum Klotho concentrations (pg/ml)				*p* value
		Q1 *N* = 2,791 (25%)	Q2 *N* = 2,793 (25%)	Q3 *N* = 2,791 (25%)	Q4 *N* = 2,794 (25%)	
Caffeine (mg/day)	120.00 (29.00–246.00)	124.00 (29.00–253.00)	133.00 (34.00–259.00)	121.00 (33.00–250.00)	102.00 (22.00–224.75)	<0.001
Ln-transformed caffeine	4.80 (3.40–5.51)	4.83 (3.40–5.54)	4.90 (3.56–5.56)	4.80 (3.53–5.53)	4.63 (3.14–5.42)	<0.001
Klotho (pg/ml)	800.70 (654.10–990.00)	554.80 (488.30–608.70)	725.50 (689.60–762.20)	884.20 (840.00–932.95)	1171.75 (1067.93–1340.33)	<0.001
Sex %						<0.001
Male	5,478 (49.05)	52.63	52.27	47.33	43.95	
Female	5,691 (50.95)	47.37	47.73	52.67	56.05	
Age (years)	58.00 (49.00–66.00)	60.00 (50.00–68.00)	58.00 (49.00–67.00)	57.00 (48.00–66.00)	56.00 (48.00–64.00)	<0.001
Race %						<0.001
Mexican American	1,703 (15.25)	15.37	15.61	15.59	14.42	
Non-Hispanic White	5,153 (46.14)	48.01	49.45	47.40	39.69	
Non-Hispanic Black	2,216 (19.84)	20.10	16.65	16.95	25.66	
Other	2,097 (18.78)	16.52	18.30	20.06	20.22	
Education %						0.006
< High school	1,337 (11.97)	12.47	12.32	11.39	11.70	
High school	4,061 (36.36)	39.02	35.55	36.30	34.57	
College or above	5,771 (51.67)	48.51	52.13	52.31	53.72	
PIR	2.66 (1.65)	2.14 (1.14–4.26)	2.31 (1.18–4.50)	2.45 (1.23–4.65)	2.27 (1.16–4.48)	0.007
BMI (kg/m^2^)	28.82 (25.30–33.30)	29.00 (25.60–33.30)	28.88 (25.35–33.30)	28.73 (25.20–33.17)	28.70 (24.93–33.47)	0.097
Alcohol consumption %						<0.001
≥ 12 drinks/year	8,000 (71.63)	76.25	72.97	71.19	66.11	
< 12 drinks/year	3,169 (28.37)	23.75	27.03	28.81	33.89	
Serum cotinine (ng/ml)	0.04 (0.01–2.39)	0.04 (0.01–33.45)	0.04 (0.01–7.09)	0.03 (0.01–0.93)	0.03 (0.01–0.69)	0.005
eGFR (ml/min/1.73 m^2^)	91.04 (75.48–102.47)	87.24 (70.24–100.19)	90.67 (75.12–102.51)	92.21 (77.20–102.75)	93.15 (79.51–103.93)	<0.001
Sedentary activity (minutes)	300.00 (180.00–480.00)	300.00 (180.00–480.00)	300.00 (180.00–480.00)	300.00 (180.00–480.00)	300.00 (180.00–480.00)	0.949
Diabetes %						0.011
Yes	1,989 (17.81)	19.78	16.76	16.19	18.50	
No	8,851 (79.25)	77.64	80.13	80.72	78.49	
Borderline	329 (2.95)	2.58	3.11	3.08	3.01	
Hypertension %						<0.001
Yes	5,238 (46.90)	51.24	45.65	45.00	45.71	
No	5,931 (53.10)	48.76	54.35	55.00	54.29	
CHD %						<0.001
Yes	585 (5.24)	6.77	5.05	4.84	4.29	
No	10,584 (94.76)	93.23	94.95	95.16	95.71	
Cancer %						0.006
Yes	1,365 (12.22)	13.65	11.89	12.68	10.67	
No	9,804 (87.78)	86.35	88.11	87.32	89.33	

Data are presented as median (IQR) for continuous variables and percentage for categorical variables. Variables were analyzed across Klotho quartiles using the Kruskal-Wallis rank sum test for continuous variables and the chi-square test for categorical variables (unweighted). Quartiles of serum Klotho concentrations (pg/ml): Q1 [151.3, 653.9], Q2 (653.9, 800.6], Q3 (800.6, 989.9], Q4 (989.9, 5038.3]. PIR, family poverty income ratio; BMI, body mass index; eGFR, estimated glomerular filtration rate; CHD, coronary heart disease.

Additionally, based on caffeine consumption quartiles (see Supplementary Table 1), significant demographic and clinical differences were observed. Individuals in the highest caffeine intake quartile (Q4) were more frequently male (57.07%) and slightly younger (median age 56 years; IQR: 48–65). A larger proportion of participants in Q4 identified as non-Hispanic White (69.41%), while lower caffeine quartiles had a higher representation of non-Hispanic Black and Mexican American individuals. Education and income levels also varied, with Q4 participants more likely to have completed college or higher education (56.78%). Higher alcohol consumption was observed in Q4, with 82.33% consuming 12 or more drinks per year. Furthermore, Q4 participants had significantly elevated serum cotinine levels (median: 0.07 ng/mL; *p* < 0.001) and a longer median sedentary time (360 min), reflecting distinct patterns in tobacco exposure and lifestyle across caffeine intake quartiles.

### 3.2 Association of caffeine consumption with serum Klotho concentrations

The negative correlation of caffeine consumption with serum Klotho concentrations is shown in Table 2. This association was evident both when caffeine was analyzed as a continuous variable (per 100 mg/day increase or ln-transformed) and when divided into quartiles, even after adjusting for confounding factors.

**Table 2:** Relationship between daily caffeine consumption and Klotho concentrations across all study population.

Daily caffeine consumption	Model 1 β (95%CI)	*p* value	Model 2 β (95%CI)	*p* value	Model 3 β (95%CI)	*p* value
Per 100 mg increase	−6.37 (−8.61, −4.14)	<0.001	−4.89 (−7.18, −2.59)	<0.001	−3.40 (−5.73, −1.07)	0.004
Ln-transformed	−8.18 (−11.16, −5.21)	<0.001	−5.82 (−8.90, −2.73)	<0.001	−3.99 (−7.11, −0.88)	0.012
Quartiles^a^						
Q1	Reference		Reference		Reference	
Q2	1.39 (−15.45, 18.23)	0.871	1.82 (−15.04, 18.68)	0.832	5.90 (−10.91, 22.70)	0.492
Q3	−30.29 (−46.63, −13.94)	<0.001	−23.72 (−40.21, −7.23)	0.005	−18.14 (−34.65, −1.62)	0.031
Q4	−42.70 (−58.41, −26.99)	<0.001	−32.66 (−48.90, −16.42)	<0.001	−23.00 (−39.41, −6.58)	0.006
P for trend		<0.001		<0.001		<0.001

Model 1: unadjusted model; Model 2: adjusted for sex, race, and age; Model 3: adjusted for sex, race, age, alcohol consumption, serum cotinine, education, PIR, BMI, eGFR, diabetes, hypertension, CHD, cancer, and sedentary activity. CI, confidence interval; PIR, family poverty income ratio; BMI, body mass index; eGFR, estimated glomerular filtration rate; CHD, coronary heart disease.

a Daily dietary caffeine consumption (mg/day) was categorized into quartiles: Q1 [0, 28] (*n* = 2,745; 25%), Q2 (28, 119] (*n* = 2,834; 25%), Q3 (119, 245] (*n* = 2,788; 25%), and Q4 (245, 4,530] (*n* = 2,802; 25%).

Each additional 100 mg of daily caffeine consumption was associated with a 6.37 pg./mL reduction in serum Klotho concentrations (95% CI: −8.61, −4.14) in the crude model (Model 1). This inverse association persisted in both Model 2 (*β* = −4.89, 95% CI: −7.18, −2.59, *p* < 0.001) and Model 3 (*β* = −3.40, 95% CI: −5.73, −1.07, *p* = 0.004), where confounders were adjusted for. Similar results were observed with ln-transformed caffeine consumption, showing a significant negative association as well.

In all three models, participants within the fourth quartile of caffeine consumption showed reductions of 42.70 pg./mL (95% CI: −58.41, −26.99), 32.66 pg./mL (95% CI: −48.90, −16.42), and 23.00 pg./mL (95% CI: −39.41, −6.58) in serum Klotho concentrations compared to those in the first quartile (P for trend <0.001).

### 3.3 Threshold effect analysis

**Figure 2:**
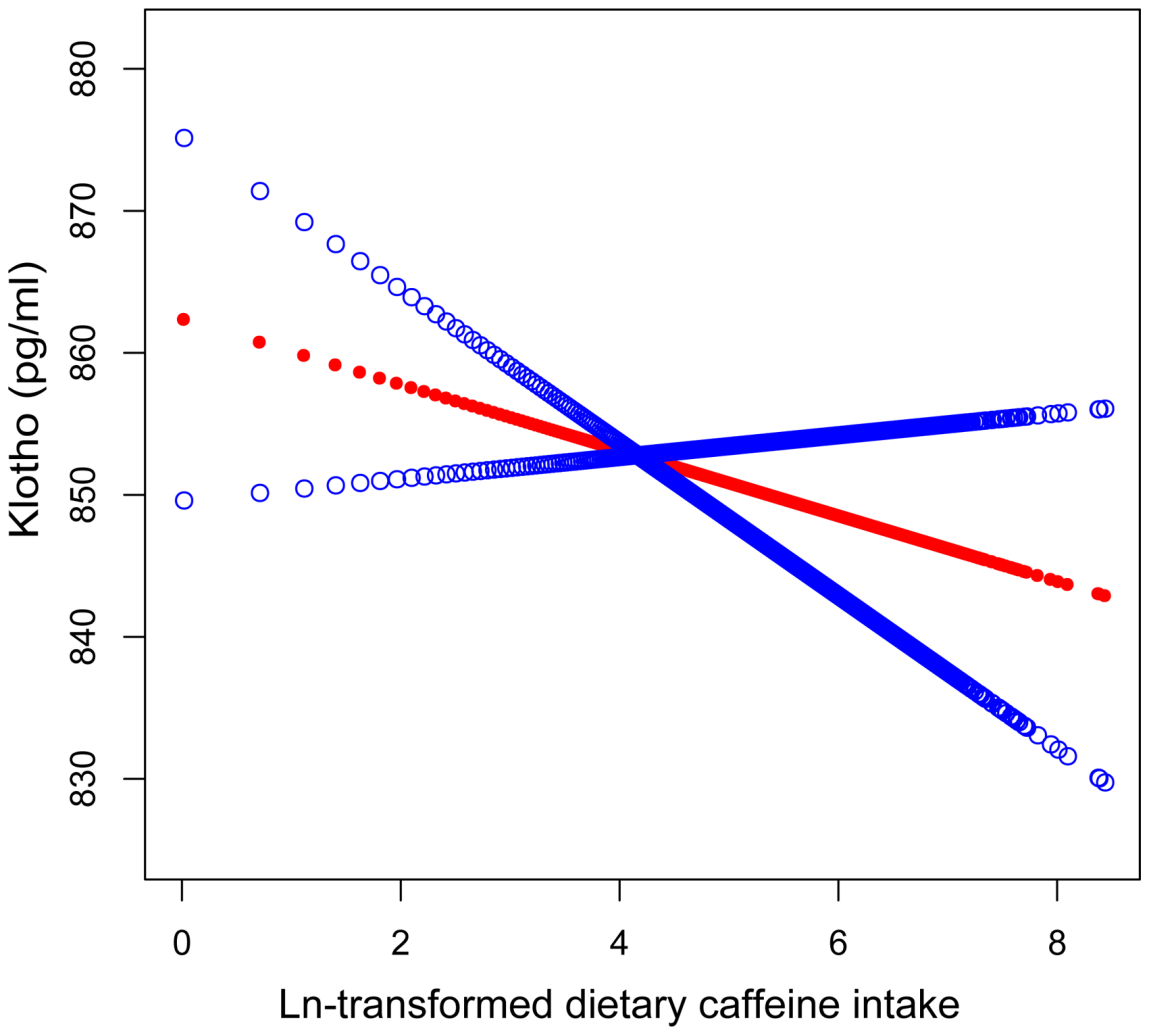
Smooth curve fitting illustrating the association between ln-transformed dietary caffeine consumption and serum Klotho concentrations. The red line denotes the fitted smooth curve representing the relationship between ln-transformed caffeine and Klotho, with the blue line indicating the 95% CI. Sex, race, age, alcohol consumption, serum cotinine, education, PIR, BMI, eGFR, diabetes, hypertension, CHD, cancer, and sedentary activity were adjusted. The *p*-value for the smooth term was 0.141, indicating no statistically significant non-linear association.

Smooth curve fitting was employed to examine the relationship between caffeine consumption and serum Klotho concentrations (Figure 2). The analysis did not provide sufficient evidence to confirm a nonlinear association, as the overall curve-fitting test did not reach statistical significance (*p* = 0.141). Threshold effect analysis (Table 3) identified a threshold point at an ln-transformed caffeine value of 3.74 (equivalent to approximately 41 mg/day). Below this point, no significant relationship was observed (*β* = 3.64, 95% CI: −2.27, 9.56, *p* = 0.228), whereas above this point, a significant negative association was detected (*β* = −14.16, 95% CI: −21.55, −6.78, *p* < 0.001). The difference in effects between the two segments was statistically significant (*p* = 0.003). The predicted Klotho concentration at the threshold point was 870.26 pg./mL (95% CI: 858.22, 882.31).

**Table 3:** Threshold effect analysis of the association between ln-transformed caffeine consumption and serum Klotho concentrations.

	Adusted β (95%CI)	*p* value
Threshold point	3.74	–
Ln-transformed caffeine <3.74	3.64 (−2.27, 9.56)	0.228
Ln-transformed caffeine >3.74	−14.16 (−21.55, −6.78)	<0.001
Difference in effects (below vs. above the threshold)	−17.80 (−29.53, −6.08)	0.003
Predicted Klotho at threshold	870.26 (858.22, 882.31)	–
Likelihood ratio test	–	0.003

Adjustment Variables: sex, race, age, alcohol consumption, serum cotinine, education, PIR, BMI, eGFR, diabetes, hypertension, CHD, cancer, and sedentary activity. CI, confidence interval; PIR, family poverty income ratio; BMI, body mass index; eGFR, estimated glomerular filtration rate; CHD, coronary heart disease.

### 3.4 Interaction test and subgroup analysis

**Figure 3:**
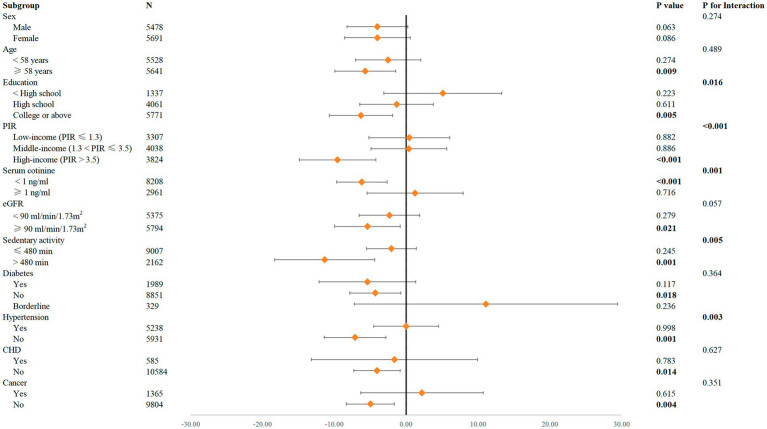
Forest plot for the impact of ln-transformed caffeine consumption on Klotho according to different variables. Sex, race, age, alcohol consumption, serum cotinine, education, PIR, BMI, eGFR, diabetes, hypertension, CHD, cancer, and sedentary activity were adjusted.

Figure 3 and Supplementary Table 2 present the results of both the interaction tests and the subgroup analyses. The interaction tests revealed significant modifications of the relationship between caffeine consumption and Klotho concentrations by variables including education, PIR, serum cotinine levels, sedentary activity, and hypertension (P for interaction <0.05). This indicates that these variables act as effect modifiers, influencing how caffeine consumption relates to Klotho levels.

In the subgroup analyses, significant negative associations between caffeine consumption and Klotho concentrations were observed among participants aged ≥58 years (*p* = 0.009), with higher education (college or above, *p* = 0.005), high income (*p* < 0.001), serum cotinine <1 ng/mL (*p* < 0.001), normal kidney function (eGFR ≥90 mL/min/1.73 m^2^, *p* = 0.021), sedentary activity >480 min daily (*p* = 0.001), and without diabetes (*p* = 0.018), hypertension (*p* = 0.001), CHD (*p* = 0.014), or cancer (*p* = 0.004). Conversely, no significant associations were observed in the sex subgroups.

## 4 Discussion

Drawing on data from a nationally representative United States sample, this study revealed a robust inverse correlation of caffeine consumption (whether analyzed continuously or by quartiles) and Klotho concentrations, remaining significant after controlling for various influencing factors. Notably, this association was more pronounced among participants with longer durations of sedentary behavior and in those without hypertension.

As far as we are aware, no research to date has specifically investigated the link of caffeine with Klotho. While this study provides evidence of an inverse association, the underlying mechanisms remain unclear. Klotho, known for its involvement in aging, is essential for regulating oxidative stress and inflammation. Caffeine, likewise, has been shown to provide health benefits, such as antioxidant, anti-inflammatory (49), and anti-aging properties. For example, research has shown that caffeine can extend lifespan in model organisms such as yeast (6) and improve both lifespan and healthspan in *Caenorhabditis elegans* (50). Animal studies have further indicated caffeine’s ability to reduce oxidative stress, neuroinflammation, and neurodegeneration (51). Additionally, coffee consumption, both regular and decaffeinated, has been linked to modulation of aging-related pathways like mTOR, which could mitigate risks of age-related diseases such as cancer (7). These findings suggest that caffeine, at moderate intake levels, may exhibit beneficial properties in biological systems.

Accumulating evidence suggests that caffeine may exert dual effects depending on its intake levels. While coffee consumption is often associated with reduced mortality risk, caffeine itself has been linked to markers of accelerated aging. Ma Jianhua et al. identified a nonlinear relationship between caffeine intake and phenotypic age, observing that moderate consumption initially reduced aging risk, but this trend reversed at higher intake levels (52). Similarly, studies examining telomere length—a recognized biomarker of cellular aging—have reported contrasting effects of caffeine and coffee. For example, Liu et al. found that higher coffee intake was associated with longer telomeres, but this relationship vanished for total dietary caffeine after controlling for coffee consumption (35). Likewise, Tucker et al. demonstrated a negative correlation between caffeine intake and telomere length, whereas coffee intake showed a positive association (36). These findings emphasize the distinct biological impacts of caffeine and coffee, suggesting that non-caffeine components of coffee, such as phenolic compounds and antioxidants (8), may mitigate caffeine’s potential harm. Supporting this, extensive prospective studies by Lopez-Garcia et al. (3) and Loftfield et al. (4) reported inverse associations between coffee consumption, including decaffeinated varieties, and all-cause mortality. Collectively, this evidence highlights caffeine’s potential toxicity at higher doses and underscores the broader protective effects of coffee, likely driven by its non-caffeine constituents.

Excessive caffeine intake, over 1 to 1.5 grams daily, has been shown to cause chronic toxicity resembling anxiety (53), and intakes above 2 grams may require hospitalization, with doses ≥5 grams potentially fatal for adults (54). This established evidence of caffeine toxicity aligns with our findings, suggesting that its impact on aging biomarkers, including Klotho, may be dose-dependent.

Our results further corroborate these insights by demonstrating a threshold effect. Klotho concentrations exhibited a significant reduction at higher caffeine intake levels, specifically above a threshold point of ln-transformed caffeine >3.74 (equivalent to ~41 mg/day). This suggests that while moderate caffeine intake may exert minimal effects or even transient benefits on Klotho levels, excessive intake appears to drive a pronounced decline. The marked difference in effects above and below this threshold supports the hypothesis of a nonlinear relationship, emphasizing that caffeine’s biological impact varies across intake levels.

Furthermore, these findings imply that previous research indicating caffeine’s antioxidant and longevity-promoting effects may reflect modest and dose-dependent benefits. Excessive caffeine intake, as observed in our study, could negate these effects, potentially leading to a reduction in Klotho levels and accelerating aging processes. This finding aligns with prior research demonstrating that caffeine can inhibit DNA repair and shorten telomeres, key biomarkers of aging. These effects, potentially mediated through pathways involving oxidative stress and inflammation, further support the hypothesis that excessive caffeine intake accelerates biological aging. In this context, caffeine’s impact on aging may differ substantially from the broader, potentially protective effects of coffee in its entirety.

These findings underscore the importance of moderating caffeine intake, particularly in individuals with higher daily consumption. Excessive intake could have adverse effects on aging-related biomarkers like Klotho, emphasizing the potential importance of tailored dietary recommendations. Further research is necessary to elucidate the complex interplay between caffeine, Klotho, and aging pathways, and to establish optimal intake levels for minimizing harm while maximizing potential benefits.

Prior research has indicated a connection between serum Klotho concentrations and both physical activity and sedentary behavior (48, 55–57). Consequently, we performed interaction tests and subgroup analyses of sedentary activity as a covariate. Interaction tests indicated that sedentary activity significantly modified the association between caffeine consumption and Klotho concentrations (P for interaction <0.05). Subgroup analyses further revealed that the negative correlation was particularly pronounced in participants sedentary for over 480 min daily. Sedentary behavior has been shown to elevate oxidative stress and systemic inflammation, both of which can inhibit Klotho expression (48, 55). In this context, caffeine may further exacerbate oxidative stress (58), potentially accelerating the decline in Klotho levels. Additionally, Geng et al. demonstrated that exercise improves metabolic disorders through increased sensitivity to FGF21, a protein related to Klotho regulation (59). Conversely, sedentary behavior could reduce FGF21 sensitivity, thereby worsening its impact on Klotho expression. Furthermore, Corrêa et al. highlighted the potential of Klotho as an ‘exerkine’—a beneficial molecule induced by exercise (57). This implies that a lack of physical activity could contribute to decreased Klotho levels in sedentary individuals. Given the strong association between sedentary behavior and chronic conditions like metabolic syndrome and inflammation, individuals with extended sedentary periods might experience greater suppression of Klotho expression, making the negative effects of caffeine more pronounced in this group. These findings suggest that individuals with prolonged sedentary behavior could be more susceptible to caffeine’s adverse impact on Klotho levels. This highlights the potential need for targeted recommendations to moderate caffeine intake in populations with high sedentary time.

Previous studies have associated serum Klotho levels with renal insufficiency (19), hypertension (40), diabetes (60), CHD (46), and cancer (47). In light of these findings, we conducted interaction tests and subgroup analyses, adjusting for covariates including eGFR, diabetes, hypertension, CHD, and cancer. The interaction tests revealed that hypertension significantly moderated the relationship between caffeine intake and Klotho levels, while no significant interaction was found for diabetes, CHD, cancer, or eGFR. In the subgroup analyses, a significant negative association between caffeine intake and Klotho levels was observed in individuals without comorbidities, whereas no such effect was evident in those with comorbidities. Hypertension, diabetes, and CHD are linked to oxidative stress and chronic inflammation, which could potentially suppress Klotho expression. Since Klotho is primarily expressed in the kidneys, renal insufficiency may significantly lower Klotho levels. Additionally, in cancer patients, tumor-related factors often modulate Klotho expression. Therefore, in individuals with comorbidities, these baseline conditions could have already reduced Klotho levels, potentially obscuring the negative effects of caffeine. Conversely, healthier individuals without comorbidities tend to exhibit higher and more variable Klotho levels, making the impact of caffeine more detectable, thus resulting in the observed significant negative correlation. The findings imply that the negative impact of dietary caffeine on Klotho could be more pronounced in healthier individuals without underlying health conditions, indicating the need for cautious recommendations regarding caffeine consumption, especially in populations without comorbidities. However, given that the interaction effects were not statistically significant across all comorbid conditions (only hypertension showed a significant interaction), these associations must be viewed with caution, particularly in view of the study’s cross-sectional design.

Furthermore, interaction tests revealed that the relationship between caffeine intake and Klotho levels was significantly influenced by socioeconomic status (education and income) and serum cotinine levels, suggesting that these factors may modulate the effect of caffeine on Klotho. Subgroup analyses further indicated that the negative association between caffeine consumption and Klotho was stronger in individuals with higher socioeconomic status and lower serum cotinine levels (< 1 ng/mL). This pattern may reflect that lifestyle factors, such as reduced tobacco exposure, allow caffeine’s effects on Klotho to become more observable, as oxidative stress from smoking might otherwise obscure these effects (61, 62). Additionally, higher socioeconomic status often correlates with healthier baseline characteristics (63, 64), making Klotho levels more responsive to dietary influences like caffeine. Therefore, these findings suggest that caffeine’s impact on Klotho could vary depending on lifestyle and socioeconomic factors, supporting the need for tailored public health recommendations based on individual health behaviors and backgrounds.

Given the widespread global consumption of caffeine, the observed association with Klotho levels could have important public health implications, particularly for aging populations, where maintaining Klotho levels may play a role in preventing age-related diseases. Further research is needed to explore whether reducing caffeine consumption helps maintain Klotho levels and improve health outcomes.

Leveraging data from the comprehensive NHANES cohort, our study is the first to explore the association of caffeine consumption with serum Klotho concentrations. However, there are multiple limitations. First, reliance on a single day of 24-h dietary recall may introduce memory-related inaccuracies, as participants may not accurately recall their intake, leading to under-or over-estimation of caffeine consumption. Additionally, a single 24-h snapshot does not capture variability in dietary patterns, such as seasonal or geographic differences, potentially reducing the generalizability of the findings. Multiple dietary recalls across different seasons could provide more reliable estimates of habitual caffeine intake and strengthen future analyses. Second, the ability to infer causality is limited by the design of the study, which is cross-sectional. Third, as the study was restricted to American adults aged 40 to 79, the applicability of the results to other populations could be limited. Finally, despite adjustments for known confounders, some unmeasured factors may still influence the results.

Future studies should prioritize investigating the long-term impact of caffeine on Klotho levels using longitudinal research designs. Additionally, investigating the underlying biological mechanisms in different population subgroups could provide deeper insights into the caffeine-Klotho relationship.

## 5 Conclusion

Our findings reveal a significant inverse association between caffeine consumption and serum Klotho concentrations in middle-aged American adults, with evidence of a dose-dependent relationship. Specifically, a threshold effect was identified, where higher caffeine intake beyond approximately 41 mg/day was associated with a more pronounced reduction in Klotho levels. Subgroup analyses indicated that this negative association was particularly significant in individuals with sedentary activity >480 min per day and without hypertension. These results underscore the necessity for additional investigation to determine how caffeine consumption impacts aging and overall health.

## Data Availability

Publicly available datasets were analyzed in this study. This data can be found at: http://www.cdc.gov/nchs/nhanes.htm.

## Footnotes

^1^ http://www.cdc.gov/nchs/nhanes.htm

^2^ https://www.kidney.org/content/ckd-epi-creatinine-equation-2021

^3^ http://www.empowerstats.com

^4^ http://www.R-project.org
