# Supplementary material for: Dietary caffeine and its negative link to serum Klotho concentrations: evidence from the National Health and Nutrition Examination Survey
Source: Front Nutr. 2024 Dec 11;11:1497224. doi: 10.3389/fnut.2024.1497224 (PMC11669319; doi:10.3389/fnut.2024.1497224)
Supplement: Supplementary file 1 [file Table_1.DOCX]

**Supplementary Table 1.** Baseline demographic and clinical characteristics of participants in the NHANES study by caffeine consumption quartiles (N = 11,169).

| Variables | All  N=11169 (100%) | Quartiles of dietary caffeine consumption (mg/day) | | | | P value |
| --- | --- | --- | --- | --- | --- | --- |
|  |  | Q1  N=2745 (25%) | Q2  N=2834 (25%) | Q3  N=2788 (25%) | Q4  N=2802 (25%) |  |
| Caffeine (mg/day) | 120.00 (29.00-246.00) | 2.00 (0.00-7.00) | 73.00 (50.00-97.00) | 172.00 (144.00-205.00) | 367.00 (294.00-504.00) | <0.001 |
| Klotho (pg/ml) | 800.70 (654.10-990.00) | 812.00 (655.70-1016.90) | 813.90 (654.70-1012.50) | 792.65 (656.35-979.42) | 787.90 (647.60-962.55) | <0.001 |
| Sex % |  |  |  |  |  | <0.001 |
| Male | 5478 (49.05) | 44.59 | 44.64 | 49.86 | 57.07 |  |
| Female | 5691 (50.95) | 55.41 | 55.36 | 50.14 | 42.93 |  |
| Age (years) | 58.00 (49.00-66.00) | 59.00 (49.00-67.00) | 58.00 (48.00-67.00) | 58.00 (49.00-66.00) | 56.00 (48.00-65.00) | <0.001 |
| Race % |  |  |  |  |  | <0.001 |
| Mexican American | 1703 (15.25) | 17.23 | 18.17 | 15.93 | 9.67 |  |
| Non-Hispanic White | 5153 (46.14) | 30.53 | 36.56 | 47.85 | 69.41 |  |
| Non-Hispanic Black | 2216 (19.84) | 33.15 | 22.23 | 16.68 | 7.53 |  |
| Other | 2097 (18.78) | 19.09 | 23.04 | 19.55 | 13.38 |  |
| Education % |  |  |  |  |  | <0.001 |
| < High school | 1337 (11.97) | 15.45 | 14.64 | 10.15 | 7.67 |  |
| High school | 4061 (36.36) | 36.98 | 37.40 | 35.51 | 35.55 |  |
| College or above | 5771 (51.67) | 47.58 | 47.95 | 54.34 | 56.78 |  |
| PIR | 2.66 (1.65) | 1.93 (1.01-3.82) | 2.15 (1.13-4.13) | 2.52 (1.29-4.77) | 2.71 (1.27-4.95) | <0.001 |
| BMI (kg/m^2^) | 28.82 (25.30-33.30) | 29.13 (25.40-33.70) | 28.70 (25.14-32.86) | 28.60 (25.30-33.10) | 28.90 (25.37-33.40) | 0.018 |
| Alcohol consumption % |  |  |  |  |  | <0.001 |
| ≥ 12 drinks/year | 8000 (71.63) | 62.81 | 67.15 | 74.10 | 82.33 |  |
| < 12 drinks/year | 3169 (28.37) | 37.19 | 32.85 | 25.90 | 17.67 |  |
| Serum cotinine (ng/ml) | 0.04 (0.01-2.39) | 0.03 (0.01-0.31) | 0.03 (0.01-0.32) | 0.03 (0.01-1.70) | 0.07 (0.01-184.75) | <0.001 |
| eGFR (ml/min/1.73  m^2^) | 91.04 (75.48-102.47) | 89.54 (73.08-101.89) | 91.64 (75.70-103.40) | 91.05 (76.12-101.89) | 91.63 (76.84-102.65) | <0.001 |
| Sedentary activity (minutes) | 300.00 (180.00-480.00) | 300.00 (180.00-480.00) | 300.00 (180.00-480.00) | 300.00 (180.00-480.00) | 360.00 (240.00-480.00) | <0.001 |
| Diabetes % |  |  |  |  |  | <0.001 |
| Yes | 1989 (17.81) | 20.66 | 17.57 | 17.22 | 15.85 |  |
| No | 8851 (79.25) | 75.85 | 79.64 | 79.99 | 81.44 |  |
| Borderline | 329 (2.95) | 3.50 | 2.79 | 2.80 | 2.71 |  |
| Hypertension % |  |  |  |  |  | <0.001 |
| Yes | 5238 (46.90) | 52.35 | 46.75 | 44.76 | 43.83 |  |
| No | 5931 (53.10) | 47.65 | 53.25 | 55.24 | 56.17 |  |
| CHD % |  |  |  |  |  | 0.944 |
| Yes | 585 (5.24) | 5.10 | 5.12 | 5.34 | 5.39 |  |
| No | 10584 (94.76) | 94.90 | 94.88 | 94.66 | 94.61 |  |
| Cancer % |  |  |  |  |  | 0.001 |
| Yes | 1365 (12.22) | 11.33 | 11.36 | 11.84 | 14.35 |  |
| No | 9804 (87.78) | 88.67 | 88.64 | 88.16 | 85.65 |  |

Data are presented as median (IQR) for continuous variables and percentage for categorical variables.

Variables were analyzed across caffeine quartiles using the Kruskal-Wallis rank sum test for continuous variables and the chi-square test for categorical variables (unweighted).

Quartiles of caffeine consumption (mg/day): Q1 [0,28], Q2 (28,119], Q3 (119,245], Q4 (245,4530].

Abbreviations: PIR: family poverty income ratio; BMI: body mass index; eGFR: estimated glomerular filtration rate; CHD, Coronary heart disease.

**Supplementary Table 2.** The relationship between dietary caffeine consumption and Klotho concentrations, grouped by sex, age, education, PIR, serum cotinine, eGFR, sedentary activity, diabetes, hypertension, CHD, cancer.

| Participants | N | Model 3  β (95%CI) | P value | P for Interaction^a^ |
| --- | --- | --- | --- | --- |
| Sex subgroup |  |  |  | 0.274 |
| Male | 5478 | -4.02 (-8.25, 0.21) | 0.063 |  |
| Female | 5691 | -4.01 (-8.59, 0.57) | 0.086 |  |
| Age subgroup |  |  |  | 0.489 |
| Age < 58 years | 5528 | -2.53 (-7.07, 2.01) | 0.274 |  |
| Age *≥* 58 years | 5641 | -5.70 (-9.95, -1.46) | **0.009** |  |
| Education subgroup |  |  |  | **0.016** |
| < High school | 1337 | 5.11 (-3.10, 13.31) | 0.223 |  |
| High school | 4061 | -1.33 (-6.46, 3.80) | 0.611 |  |
| College or above | 5771 | -6.30 (-10.70, -1.90) | **0.005** |  |
| PIR subgroup |  |  |  | **<0.001** |
| Low-income (PIR ≤ 1.3) | 3307 | 0.43 (-5.21, 6.07) | 0.882 |  |
| Middle-income (1.3 < PIR ≤ 3.5) | 4038 | 0.38 (-4.88, 5.65) | 0.886 |  |
| High-income (PIR > 3.5) | 3824 | -9.57 (-14.91, -4.23) | **<0.001** |  |
| Serum cotinine subgroup |  |  |  | **0.001** |
| S-cotinine < 1ng/ml | 8208 | -6.19 (-9.70, -2.67) | **<0.001** |  |
| S-cotinine ≥ 1ng/ml | 2961 | 1.24 (-5.45, 7.93) | 0.716 |  |
| eGFR subgroup |  |  |  | 0.057 |
| eGFR < 90ml/min/1.73m^2^ | 5375 | -2.33 (-6.56, 1.89) | 0.279 |  |
| eGFR ≥ 90ml/min/1.73m^2^ | 5794 | -5.39 (-9.96, -0.82) | **0.021** |  |
| Sedentary activity subgroup |  |  |  | **0.005** |
| Sedentary activity ≤ 480min | 9007 | -2.07 (-5.55, 1.42) | 0.245 |  |
| Sedentary activity > 480min | 2162 | -11.35 (-18.32, -4.38) | **0.001** |  |
| Diabetes subgroup |  |  |  | 0.364 |
| Yes | 1989 | -5.39 (-12.14, 1.35) | 0.117 |  |
| No | 8851 | -4.30 (-7.86, -0.73) | **0.018** |  |
| Borderline | 329 | 11.11 (-7.23, 29.44) | 0.236 |  |
| Hypertension subgroup |  |  |  | **0.003** |
| Yes | 5238 | -0.01 (-4.52, 4.50) | 0.998 |  |
| No | 5931 | -7.12 (-11.43, -2.81) | **0.001** |  |
| CHD subgroup |  |  |  | 0.627 |
| Yes | 585 | -1.63 (-13.20, 9.94) | 0.783 |  |
| No | 10584 | -4.06 (-7.29, -0.83) | **0.014** |  |
| Cancer subgroup |  |  |  | 0.351 |
| Yes | 1365 | 2.19 (-6.34, 10.72) | 0.615 |  |
| No | 9804 | -4.97 (-8.32, -1.62) | **0.004** |  |

Model 3 was adjusted for the following covariates: sex, age, race, education, PIR, alcohol consumption, eGFR, BMI, hypertension, serum cotinine, diabetes, CHD, cancer, and sedentary activity. The subgroup variable was not considered in the subgroup analysis.

^a^ P value for the interaction of dietary caffeine consumption with the following covariates: sex, age, race, education, PIR, alcohol consumption, eGFR, BMI, hypertension, serum cotinine, diabetes, CHD, cancer, and sedentary activity. The subgroup variable was not considered in the interaction test.

Dietary caffeine consumption was natural log-transformed for analysis as a continuous variable.

Abbreviations: CI: confidence interval; PIR: family poverty income ratio; BMI: body mass index; eGFR: estimated glomerular filtration rate; CHD, Coronary heart disease.

Bold indicates significance with p < 0.05.
